# Double Decomposition and Fuzzy Cognitive Graph-Based Prediction of Non-Stationary Time Series

**DOI:** 10.3390/s24227272

**Published:** 2024-11-14

**Authors:** Junfeng Chen, Azhu Guan, Shi Cheng

**Affiliations:** 1 College of Artificial Intelligence and Automation, Hohai University, Changzhou 213200, China; 2 Jiangsu Key Laboratory of Power Transmission & Distribution Equipment Technology, Hohai University, Changzhou 213200, China; 3 College of Information Science and Engineering, Hohai University, Changzhou 213200, China; guanazhu@163.com; 4 School of Computer Science, Shaanxi Normal University, Xi’an 710119, China; cheng@snnu.edu.cn

**Keywords:** non-stationary time series prediction, high-order cognitive fuzzy map, wavelet decomposition, empirical mode decomposition, ridge regression

## Abstract

Deep learning models, such as recurrent neural network (RNN) models, are suitable for modeling and forecasting non-stationary time series but are not interpretable. A prediction model with interpretability and high accuracy can improve decision makers’ trust in the model and provide a basis for decision making. This paper proposes a double decomposition strategy based on wavelet decomposition (WD) and empirical mode decomposition (EMD). We construct a prediction model of high-order fuzzy cognitive maps (HFCM), called the WE-HFCM model, which considers interpretability and strong reasoning ability. Specifically, we use the WD and EDM algorithms to decompose the time sequence signal and realize the depth extraction of the signal’s high-frequency, low-frequency, time-domain, and frequency domain features. Then, the ridge regression algorithm is used to learn the HFCM weight vector to achieve modeling prediction. Finally, we apply the proposed WE-HFCM model to stationary and non-stationary datasets in simulation experiments. We compare the predicted results with the autoregressive integrated moving average (ARIMA) and long short-term memory (LSTM) models.For stationary time series, the prediction accuracy of the WE-HFCM model is about 45% higher than that of the ARIMA, about 35% higher than that of the SARIMA model, and about 16% higher than that of the LSTM model. For non-stationary time series, the prediction accuracy of the WE-HFCM model is 69% higher than that of the ARIMA and SARIMA models.

## 1. Introduction

Time series forecasting can help enterprises and governments to make reasonable economic decisions and is widely used in various fields. For example, in the economic field, it can be used to predict future sales, stock prices, economic growth rates, etc. [1]. A time series forecasting model in meteorology can predict future temperature, rainfall, and other meteorological conditions to help people make reasonable travel and production plans [2]. In transportation, it can be used to predict future traffic flow and help traffic management departments carry out traffic scheduling and planning [3]. The time series prediction model in the energy field can predict elements of future power generation, such as power consumption, and provide a reasonable basis for decisions in power-dispatching departments-making [4,5,6]. In addition, time series prediction models have been widely used in medicine [7,8], tourism [9], industry [10], and other fields. Therefore, the study of time series prediction is of great significance.

Time series can be divided into stationary time series and non-stationary time series. The observed values of stationary series fluctuate at a fixed level. Although the fluctuation degree differs in different periods, there is no specific rule, and the fluctuation can be regarded as random. A non-stationary sequence is a sequence of trends, seasonality, or periodicity, which may contain only one or a combination of several components [11]. Ghaderpour et al. [12] proposed the mathematical derivation of the underlying probability distribution function for the normalized least squares wavelet spectrogram. It can simultaneously estimate trend and seasonal components in the time series, considering their correlation, which significantly improves the component estimation. Baidya et al. [13] proposed a novel TSF model designed to address the challenges posed by real-life data, delivering accurate forecasts in both multivariate and univariate settings. In real life, the non-stationary sequence is the majority, and the smoothness of the sequence is an essential premise for time series prediction modeling. However, dealing with large-scale, non-stationary time series with changing trends and rapid changes is still challenging. Explaining the potential features of non-stationary time series and their correlation is still a complex problem.

In recent years, because of the rapid development of deep learning, deep learning has also been widely used in various fields of time series prediction. Using the black box theory, researchers have developed several time series prediction models with excellent performance; examples include a time series prediction model based on long short-term memory (LSTM) [8,14], a time series prediction model based on convolutional neural network (CNN) [15], and a time series prediction model based on Transformer [16]. Li et al. [17] converted time series values into images, which were then predicted as inputs to the CNN. Nie et al. [18] used a CNN to predict the residual life of rolling bearings and proved that the proposed model is superior to the traditional model in experiments. This is because CNN results in information loss when there are too many layers. Zan et al. [19] used the TCN-TPA prediction model to predict fusion meteorological data, avoiding the problems of gradient disappearance and gradient explosion. Some researchers have applied artificial neural networks to traffic flow prediction [20] and room temperature prediction [21]. Compared with the traditional time series prediction model, the time series prediction model based on deep learning can mine more feature information. However, it has the characteristics of a black box and a long training time, which makes it difficult for people to understand. Therefore, designing a model with interpretability and good prediction accuracy is of great importance.

Some scholars have proposed that fuzzy cognitive maps (FCMs) [22] can be applied to time series prediction. FCMs have a strong ability to carry out fuzzy reasoning and semantic understanding; they are a powerful tool for constructing interpretable time series prediction models. The nodes of FCMs represent events, features, or targets; the connections between nodes represent relationships; and the values of nodes and the connections between nodes can be represented as fuzzy values. Because of their interpretability and causal reasoning ability, FCMs have been widely used in time series prediction in recent years. Stach et al. [23] used real-coded genetic algorithms to learn fuzzy cognitive maps and make numerical and linguistic predictions of time series. In order to improve the prediction accuracy of the FCM model, Lu et al. [24] designed a high-order fuzzy cognitive map to model and predict time series. In order to solve the problems of low precision, sensitivity to hyperparameters, and unrobust prediction results of time series based on FCMs, Jin et al. [25] proposed an SO_2_ concentration prediction method based on EMD and LSTM. The results show that EMD can effectively reduce the non-stationarity of the original time series and improve prediction accuracy. Some scholars have also proposed dealing with non-stationary time series using wavelet transform. Aussem et al. [26] proposed a wavelet-based feature decomposition strategy and combined it with recurrent neural network financial forecasting.

In order to construct an interpretable model and to solve the problem of ignoring the frequency-domain features in time series data, in this paper, we apply wavelet decomposition (WD), EMD, and HFCM to time series prediction. The high-frequency component of the wavelet decomposition is still non-stationary. Since empirical mode decomposition (EMD) can reduce the non-stationarity of the sequence, EMD is used to decompose the high-frequency components after wavelet decomposition. Finally, the low-frequency component of wavelet decomposition and the high component of empirical mode decomposition are taken as the input of HFCM, and the ridge regression algorithm optimizes the parameters. We call this model WE-HFCM. Using the ridge regression algorithm to learn HFCM weights, this algorithm can effectively optimize model parameters when dealing with large-scale time series. Therefore, WE-HFCM can be effectively applied to large-scale time series with hundreds or thousands of data points. Finally, to show the prediction model’s performance, this paper compares the performance of this model with the classical time series prediction model, autoregressive integrated moving average (ARIMA), and the deep learning model, LSTM, on two non-stationary time series datasets and two stationary time series datasets.

The main contributions are as follows:

(1) We design a double decomposition stage, which extracts the low-frequency and high-frequency features of the time series by wavelet decomposition and EMD decomposition and smoothes the non-stationary time series.
(2) We construct a WE-HFCM model to increase the interpretability of the model. By aggregating the eigenvalues of different frequencies and making better use of the critical information of the potential features of time series, the representation learning of node relations is realized, and the high-order fuzzy cognitive map (HFCM) is constructed for prediction.
(3) Based on the comparison and ablation experiments, the proposed method can better predict the non-stationary univariate time series.

## 2. Materials and Methods

### 2.1. Datasets

We select benchmark time series with different statistical characteristics from different fields to test the validity of the proposed model. Non-stationary time series include stock opening price (open-price) and wind speed (wind-speed) datasets. The open-price dataset recorded the opening price of stocks every day from 25 November 2015 to 17 November 2017, with a total of 500 data pieces. The wind-speed dataset is used by this research group to participate in a competition, recording the wind speed of a place from 2 January 2021 to 11 January 2021 every 15 min, with a total of 1000 data pieces. The stationary time series includes sunspots and daily minimum temperature datasets (min-temp). Sunspot records a time series of annual sunspots from 1700 to 1987, with 289 observations. Min-temp contains 800 data items. The three datasets, open-price, sunspot, and min-temp, are available on Baidu’s website.

### 2.2. Wavelet Decomposition

**Figure 1:**
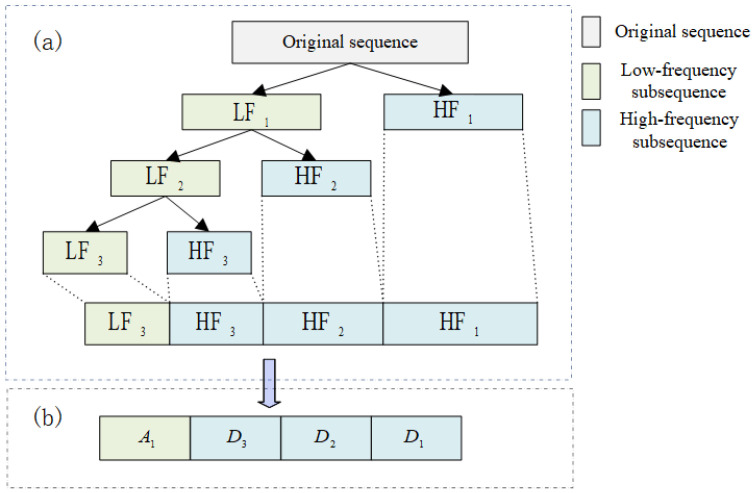
Wavelet decomposition and reconstruction: (**a**) wavelet decomposition (**b**) wavelet reconstruction.

WD decomposes the original signal into high-frequency (HF) and low-frequency (LF) components through wavelet basis functions. HF represents changes in the details of the original signal data, and LF represents the overall trend of the original signal data. The decomposition and refactoring process is shown in Figure 1. The corresponding fast algorithm in wavelet decomposition reconstruction is called the Mallat algorithm [27], expressed as follows:

(1) $$\left\{\begin{array}{c} {\mathrm{LF}}_{i+1}\left(t\right)=d\times C_{i}\left(t\right)\\ {\mathrm{HF}}_{i+1}\left(t\right)=g\times C_{i}\left(t\right) \end{array}\right.$$

where i=0,1,2,…,N. *d* is a low-pass filter for low-frequency signals. *g* is a high-pass filter for high-frequency signals. *N* is the number of decomposition layers. *t* is the time point. $C_{0}\left(t\right)$ is the original time series of the input.

The wavelet coefficients and the length of the component after passing WD are inconsistent and do not have the characteristics of the actual sequence, so the component needs to be reconstructed and solved, as shown in Figure 1a.

(2) $$C_{N}=d^{*}\times A_{d+2}+g^{*}\times D_{d+1}$$

where d=N−1,N−2,…,0. $d^{*}$ and $g^{*}$ are the dual operators of *d* and *g*, respectively.

The reconstructed time series $C_{0}^{\prime }$ is expressed as follows:

(3) $$C_{0}^{\prime }=C_{N}+D_{1}+D_{2}+\ldots +D_{N}$$

### 2.3. EMD

EMD [28] is a powerful tool that decomposes a time series into intrinsic mode functions (IMFs) and residuals. This method, which arranges the details of the time series in sequence from high frequency to low frequency, has clear advantages in handling unstable and aperiodic signals. These advantages include that the maximum difference between the number of extreme points of a local signal and the number of zeros is one and that there is symmetry between the upper and lower envelope of every part of the curve.

Wavelet decomposition (WD) plays a crucial role in generating low-frequency *A* and high-frequency *D*. Since the high-frequency part is still a non-stationary sequence, it is summated and denoted $C_{d}\left(t\right)$. We perform EMD decomposition for $C_{d}\left(t\right)$ [20,29] as follows.

(1) We interpolate the time series $C_{d}\left(t\right)$ with cubic splines and connect the extreme points to form the upper and lower envelope $e_{min}\left(t\right)$ and $e_{max}\left(t\right)$. The average envelope $m_{t}$ is calculated as in (4).
(3) The intrinsic mode function (IMF)$d_{1}$ is defined as the difference between the time series $C_{d}\left(t\right)$ and the mean envelope $m_{t}$, as shown in (5).
(3) The component of the maximum frequency of time series $C_{d}\left(t\right)$ is determined as $c_{i}$, (i=1,2,…,n), and separated from $C_{d}\left(t\right)$, as shown in Equation (6). We continue the decomposition with $r_{1}$ as input. The complete decomposition formula is shown in (7).

(4) $$m\left(t\right)=\left(e_{min}\left(t\right)+e_{max}\left(t\right)\right)/2$$

(5) $$d_{1}=C_{d}\left(t\right)-m\left(t\right)$$

(6) $$r_{1}=C_{d}\left(t\right)-c_{1}$$

(7) $$C_{d}\left(t\right)=\sum_{i=1}^{n}d_{i}\left(t\right)+r_{n}$$

where $C_{d}\left(t\right)$ is the sum of the high-frequency part of the wavelet decomposition sequence, $m\left(t\right)$ is the average of the upper and lower envelope of the extreme point, $d_{i}\left(t\right)$ is the decomposed $IMF_{i}$, $d_{1}$ is $IMF_{1}$, $c_{1}$ is the maximum frequency of the input sequence, $r_{1}$ is the $C_{d}\left(t\right)$ sequence with the $c_{1}$ part removed, and $r_{n}$ is the residual term.

### 2.4. FCMs

FCMs [22] are developed by Kosko based on Axelord cognitive maps, extending the ternary relation between concepts (1, 0, 1) to the fuzzy relation on the interval [−1, 1]. This makes the FCMs more informative. FCMs are a graph structure that connect causal events, participation values, goals and trends in a fuzzy feedback dynamic system through arcs between concepts. The nodes are concepts, entities, etc., and the arcs represent causal relationships between concepts or entities. The degree of causal influence can be expressed by fuzzy values [0, 1] or described by natural language, such as weak, very weak, medium, and strong.

The semantics contained in standard FCMs are represented by a 4-tuple $\left(C,\;W,\;A,\;f\right)$. This representation is a crucial aspect of FCM, which consists of *n* nodes, $X=\left\{X_{1},X_{2},\ldots ,X_{n}\right\}$ is a set of *n* nodes, and *W* is a weight matrix of n×n dimensions:

(8) $$W=\left(\begin{array}{ccc} W_{11} & \cdots & W_{1n}\\ \vdots & \ddots & \vdots \\ W_{n1} & \cdots & W_{nn} \end{array}\right)$$

The status value of the $X_{i}$ node at time t+1 is affected by the status value and weight value of all nodes connected to it at time *t*. In this paper, *X* is the $d_{i}\left(t\right)$ set obtained by decomposing the low-frequency component A in WD and EMD. The state value of the $X_{i}$ node at time t+1 can be expressed by (9).

(9) $$A_{i}\left(t+1\right)=f\left(\sum_{j=1}^{n}w_{ji}A_{i}\left(t\right)\right)$$

where $A_{i}\left(t\right)$ and $A_{i}\left(t+1\right)$ represent the status value of node $X_{i}$ at time *t* and time t+1, respectively, $t=\left\{1,2,3,\ldots ,T\right\}$. *f* is the activation function.

(10) $$\left\{\begin{array}{cc} \;w_{ji}>0 & \\ \;w_{ji}=0 & ,w_{ji}\in \left[-1,\;1\right]\\ w_{ji}<0 \end{array}\right.$$

The weight $w_{ji}$ in (10) can reflect the degree and direction of causal influence between node $X_{i}$ and node $X_{j}$. If $w_{ji}>0$, the two nodes are positively correlated. If $w_{ji}<0$, the two nodes are negatively correlated. If $w_{ji}=0$, there is no relationship between the two nodes.

The current state of the FCM is not only determined by the state of the previous moment but also affected by the state of the past. Stach et al. [30] introduced higher-order state values into the FCM model to enhance its approximation ability. The calculation of the HFCM of order *k* is shown in (11).

(11) $$A_{i}\left(t+1\right)=f\left(\sum_{j=1}^{n}\left(w_{ji}^{1}A_{j}\left(t\right)+w_{ji}^{2}A_{j}\left(t-1\right)+\cdots +w_{ji}^{k}A_{j}\left(t-k+1\right)\right)+w_{i0}\right)$$

where $w_{ji}^{k}$ represents the relation of the *j*-th node to the *i*-th node at the time step and is the constant deviation of the *i*-th node relative to the 0-th node.

**Figure 2:**
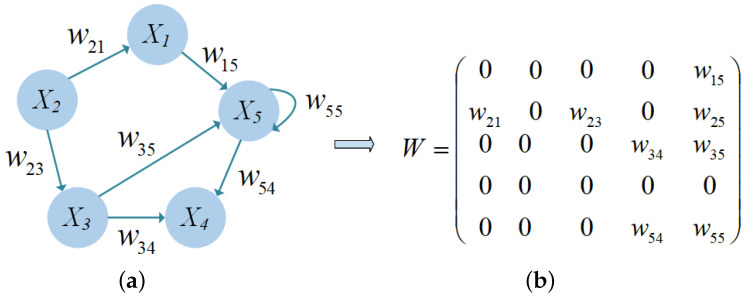
(**a**) Fuzzy cognitive map with five nodes. (**b**) The weight matrix of FCM.

Figure 2 shows a fuzzy cognitive graph with five nodes and a weight matrix. A one-way arrow pointing from $X_{2}$ to $X_{1}$ indicates that node $X_{2}$ is correlated with node $X_{1}$, and its weight is $X_{21}$.

### 2.5. WE-HFCM Prediction Model

The HFCM’s node and relationship weights are two key factors. Obtaining meaningful nodes and a node relation matrix is a challenging research problem. The univariate time series, being a one-dimensional numerical series, cannot directly form the multi-node structure of HFCM. This paper proposes a double decomposition strategy based on WD and EMD to solve the time series prediction problem through the HFCM framework. The resulting WE-HFCM framework has the potential to impact the field of time series prediction significantly.

**Figure 3:**
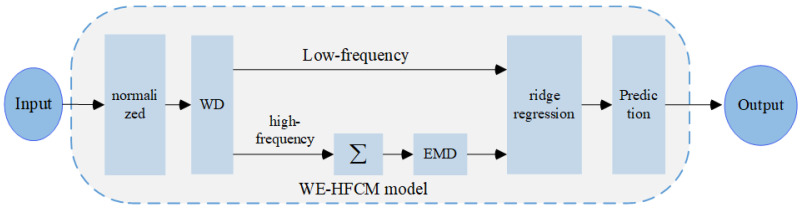
Basic framework of the WE-HFCM prediction model.

Figure 3 illustrates the meticulous process of time series prediction using WE-HFCM. The original time series is first normalized, and the numerical data are transformed into HFCM nodes through WD and EMD decomposition. WD decomposes the time series into two parts, the low-frequency component and high-frequency component, and EMD further decomposes the high-frequency component into multiple IMFs through accumulation. The input of HFCM is the set of low-frequency components and multiple IMFs. The weight matrix of HFCM is then optimized using the rigorous ridge regression algorithm. Finally, in each time step, the values of all nodes are summed, and the predicted value is output. This comprehensive process ensures the reliability of the WE-HFCM model.

#### 2.5.1. Double Decomposition of Time Series

**Figure 4:**
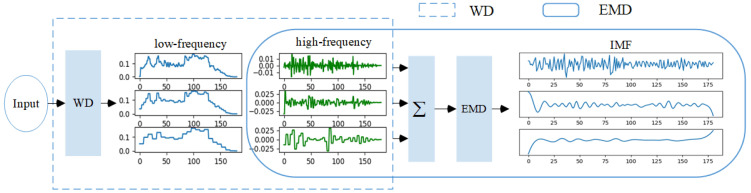
Double decomposition frame.

Wavelet decomposition provides signal analysis in time and frequency, allowing decision makers to observe time series at different resolution levels. However, there are still non-stationary subsequences in the high-frequency components obtained by wavelet decomposition, and EMD decomposition has apparent advantages in dealing with unstable and non-periodic signals. Therefore, this paper introduces EMD to further decompose the high-frequency part of WD decomposition. The double decomposition of the time series is shown in Figure 4. We first normalize the input time series and map it to the range [−1, 1]. Then, through discrete wavelet transformation and reconstruction, the normalized time series is decomposed into low-frequency component A and high-frequency component D by (1) and (2). The low-frequency component represents the overall trend information of the input time series, and the high-frequency part represents the detailed information of the input time series. As far as we know, previous studies only focus on the low-frequency component, ignoring the high-frequency information in the time series. Therefore, this paper sums up the high-frequency components and decomposes them through EMD to obtain multiple IMFs, reflecting the time series’ intrinsic characteristics.

#### 2.5.2. Ridge Regression for Learning HFCM

Ridge regression is a regularization method of linear regression, which has a good advantage in dealing with datasets with correlation between predictors [31]. Ridge regression works to add a penalty term based on the least squares method and impose a penalty by adjusting the size of the coefficient to solve the shortcomings of linear regression.

**Figure 5:**
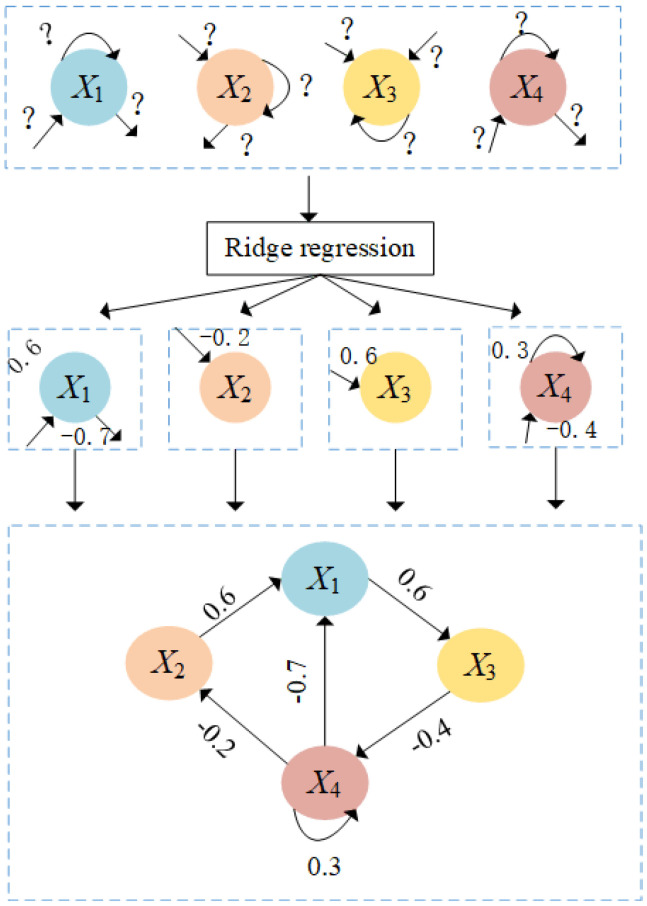
The learning process of HFCM with four node.

The multivariate time series obtained from wavelet decomposition and EMD decomposition are used to learn the weight matrix of HFCM. In the following, we use second-order HFCM as an example to illustrate how to learn HFCM weights by ridge regression. The proposed method can be easily extended to HFCM learning in any order. Wu et al. [32] pointed out that the problem of learning the weight matrix for FCM can be reduced to the problem of learning the local connections of the nodes separately. As shown in Figure 5, different spheres represent different nodes, we also use the same decomposition strategy in the HFCM learning method. Firstly, a subnetwork is constructed between node i and its neighboring nodes, and the HFCM learning problem with four nodes is decomposed into four subproblems, one for each subnetwork. The modeling of each subproblem is essentially a signal reconstruction problem, including the difference between the available sequence and the generated sequence and the sparse structure from all nodes to a specific node. Each subproblem is optimized by ridge regression. Taking node $X_{2}$ as an example, we use a lasso to learn the structure of node $X_{2}$ from nodes $X_{1}$, $X_{2}$, $X_{3}$, and $X_{4}$ and return the relationship from node $X_{2}$ to node $X_{1}$ with $W_{21}=0.68$. Finally, after learning all nodes’ neighboring nodes, we combine the local connections into the whole HFCM.

The nonlinear dynamic equation of HFCM is linearized by inverse transformation, expressed as

(12) $$\varphi^{-1}\left(A_{i}\left(t+1\right)\right)=\sum_{j=1}^{n}\left(w_{ij}^{1}A_{j}\left(t\right)+w_{ij}^{2}A_{j}\left(t-1\right)+w_{i0}\right)$$

where $\varphi^{-1}$ is the inverse of the transfer function φ. Once a time series of length *L* at different time steps *t* is available, the transformed dynamic Equation (12) can be rewritten in vector form.

(13) $$Y_{i}=XW_{i}$$

where $Y_{i}$ is the vector containing $\varphi^{-1}\left(A_{i}\left(t+1\right)\right)$, *X* is the state matrix of all states $A_{j}\left(t\right)$ of different *t* nodes, and $w_{i}$ is the weight vector between all nodes and the *i*-th node. Equations (14)–(16) show the three variables’ expression.

(14) $$Y_{i}=\left[\begin{array}{c} \varphi^{-1}\left(A_{i}\left(3\right)\right)\\ \varphi^{-1}\left(A_{i}\left(4\right)\right)\\ \vdots \\ \varphi^{-1}\left(A_{i}\left(5\right)\right) \end{array}\right]$$

(15) $$W_{i}^{T}=\left[\begin{array}{cccccccc} w_{i1}^{1} & w_{i1}^{2} & w_{i2}^{1} & w_{i2}^{2} & \cdots & w_{iN_{c}}^{1} & w_{iN_{c}}^{2} & w_{i0} \end{array}\right]$$

(16) $$X=\left[\begin{array}{cccc} A_{1}\left(2\right) & \cdots & A_{N_{c}}\left(1\right) & 1\\ A_{1}\left(3\right) & \cdots & A_{N_{c}}\left(2\right) & 1\\ \vdots & \vdots & \vdots & \vdots \\ A_{1}\left(L-1\right) & \cdots & A_{N_{c}}\left(L-1\right) & 1 \end{array}\right]$$

Ridge regression is used to solve the following optimization problems, improve the generalization ability of the WE-HFCM model, and determine the local connection of the *i*-th node.

(17) $$\min_{W_{i}}\left\{\left(1/2L\right){∥Y_{i}-XW_{i}∥}_{2}^{2}+\partial {∥W_{i}∥}_{2}\right\}$$

where ${∥W_{i}∥}_{2}=\sqrt{\sum_{k}W_{ik}^{2}}$. *∂* is a regularized parameter that is generally non-negative. The greater the value of *∂*, the greater the shrinkage and the stronger the model’s robustness to collinearity. And *k* denotes a higher-order fuzzy cognitive map of order *k*. Equation (17) determines the weight vector $W_{i}$ between all nodes and the i-th node. We used ridge regression [33] from the Python library Scikit-learn to learn the weight vector.

### 2.6. Data Preprocessing and Evaluation Indicators

#### 2.6.1. Data Preprocessing

The amplitude signal of the time series is used as input to the proposed model, and we need to normalize it. This paper uses the Min-Max normalization method to normalize the input time series and map it uniformly to the range [−1, 1]. The maximum and minimum values of the original time series are set to $X_{max}$ and $X_{min}$, respectively, and the maximum and minimum values of the normalized time series are $X_{high}$ and $X_{low}$, respectively. We normalize the time series from range [$X_{min}$, $X_{max}$] to range [$X_{low}$, $X_{high}$ ] by (18).

(18) $$\bar{X}=\left(X-X_{min}\right)\left(X_{high}-X_{low}\right)/\left(X_{max}-X_{min}\right)+X_{low}$$

Here, *X* represents the original time series, and $\bar{X}$ represents the normalized time series.

This paper divides the normalized time series data into three subsets: training set, validation set, and test set, as shown in Table 1. The training dataset is used to learn the weight matrix of the HFCM prediction model, the validation dataset is used to select the best model, and the test dataset is employed to evaluate the prediction accuracy.

#### 2.6.2. Evaluation Indicators

In this paper, three evaluation criteria are used to evaluate the performance of the method: Root Mean Squared Error (RMSE), Mean Absolute Error (MAE), and Mean Absolute Percentage Error (MAPE). MAE and RMSE measure the absolute value of the predicted deviation from the actual value. MAE does not consider the positive or negative of the predicted value and pays more attention to the size of the absolute error. RMSE is easy to understand, convenient to calculate, and sensitive to outliers. The MAPE measures the relative magnitude (i.e., percentage) by which the predicted values deviate from the actual values. MAE and MAPE are relatively less susceptible to extreme values. However, RMSE uses the square of the error, amplifying the prediction error, making it more sensitive to outlier data, and highlighting the error values with significant influence. The smaller the values of these criteria, the closer the prediction results are to the original time series values. The evaluation formula is defined as follows.

(19) $$\mathrm{RMSE}=\left(\sqrt{\sum_{i=1}^{L}\left(X_{i}-\bar{X_{i}}\right)}\right)/L^{2}$$

(20) $$\mathrm{MAE}=\left(\sum_{i=1}^{L}\left|X_{i}-\bar{X_{i}}\right|\right)/L$$

(21) $$\mathrm{MAPE}=\left(\sum_{i=0}^{L}\left|\left(X_{i}-\bar{X_{i}}\right)/X_{i}\right|\right)/L$$

where *L* is the time series length, and $X_{i}$ and $\bar{X_{i}}$ represent the normalized original and predicted time series.

## 3. Result and Analysis

This section explores the influence of different parameter values on the prediction performance of the WE-HFCM model and comprehensively evaluates its prediction performance.

### 3.1. Model Parameters

To verify the prediction effect of the model proposed in this paper, the WE-HFCM model was compared with the ARIMA and LSTM models. The model parameters are shown in Table 2.

**Figure 6:**
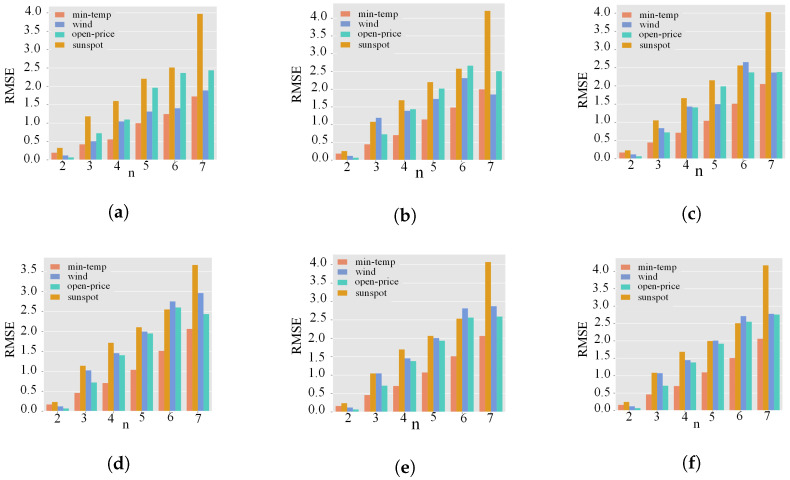
The RMSE values of HFCM of 2–7 nodes in the four datasets correspond to the order 1–6 HFCM, respectively. (**a**) k = 1, (**b**) k = 2, (**c**) k = 3, (**d**) k = 4, (**e**) k = 5, (**f**) k = 6.

For different datasets, the optimal parameters of the model are different. The regularized parameter *∂* is the best value selected by cross-validation, here $\partial =1\times 10^{-12}$. Figure 6 discusses the error comparison of the wavelet decomposition of 2 to 7 times, respectively, in the four datasets’ second to seventh-order HFCM model. From Figure 6, we can see that for the sunspot dataset, the optimal model parameters of HFCM are k=4 and n=2. For the min-temp dataset, the optimal model parameters of HFCM are k=5 and n=2. For the wind-speed and open-price datasets, the optimal model parameters of HFCM are k=2 and n=2. Generally, the order of the optimal parameters of the HFCM model on non-stationary datasets is lower than that on stationary data sets.

### 3.2. Analysis of Experimental Results

**Figure 7:**
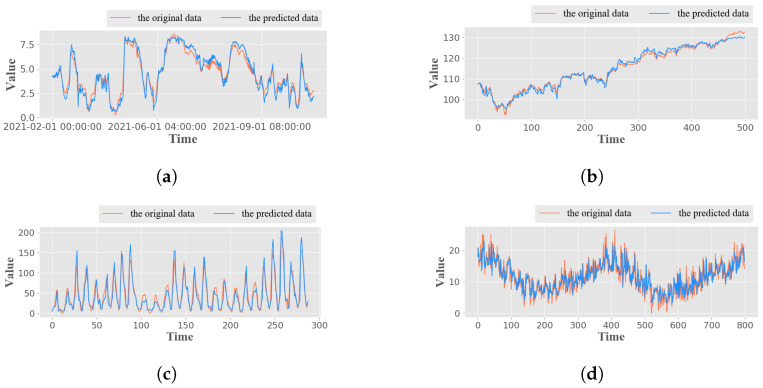
WE-HFCM predictions on four datasets: (**a**) wind-speed, (**b**) open-price, (**c**) sunspot, (**d**) min-temp.

We meticulously tested the proposed model on four datasets, comparing the original and predicted data. Following the model parameter setting in Section 3.1 we determined the optimal WE-HFCM model parameters for each dataset. The prediction results of the WE-HFCM model on the four datasets are presented in Figure 7. Figure 7a,b show the prediction results of the proposed WE-HFCM model in non-stationary time series, and Figure 7c,d show the prediction results of the WE-HFCM model in stationary time series. As seen in Figure 7, the model’s prediction results follow the data trend and are accurate for non-stationary and stationary time series.

In order to better reflect the effectiveness of the WE-HFCM model proposed in this paper, it is compared with the classical time series prediction model ARIMA, SARIMA and the deep learning model LSTM, and the results are shown in Table 3. The best-performing result is indicated by bold skew, and the second best-performing result is indicated by skew. This means that the model with the lowest error is considered the best-performing model, and the second-lowest error is considered the second-best-performing model. The experimental results show that on non-stationary datasets, the error of the WE-HFCM model is much smaller than that of the ARIMA model. However, the prediction results are slightly inferior to that of the LSTM prediction model. The prediction errors of the WE-HFCM model on the stationary time series are higher than those of the non-stationary time series. However, compared with the ARIMA and LSTM models, the prediction errors of the WE-HFCM model are lower.

RSME values, i.e., the root mean square error values, measure the differences between values predicted by a model and observed values. The error value of the ARIMA model is about two times that of the WE-HFCM, and the error value of the LSTM model is equal to that of the WE-HFCM. In the final ablation experiment, the proposed model combines wavelet decomposition and empirical mode decomposition to extract features. In order to verify the effectiveness of dual decomposition, the RMSE values of the proposed model WE-HFCM and the model Wave-HFCM that only uses wavelet decomposition for feature decomposition are compared, as shown in Table 4. It can be seen that the error values of the proposed model WE-HFCM are all smaller than those of the single decomposition model Wave-HFCM, which verifies that the use of double decomposition can better improve the prediction accuracy of the model. Table 5 records the RMSE of WE-HFCM on all datasets, i.e., the training dataset, validation dataset, and test dataset, for each time series.

**Figure 8:**
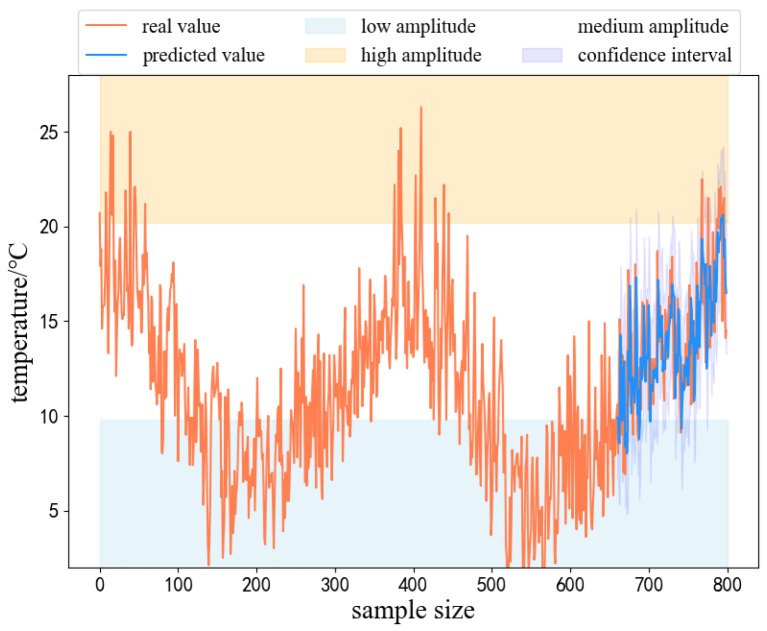
WE-HFCM interpretation analysis on the min-temp test dataset.

In order to reflect the interpretability of the WE-HFCM model proposed in this paper, the min-temp dataset is taken as an example for analyzing the interpretability of the model. The minimum, median, and maximum values of the min-temp time series are selected as three fuzzy variables, and their corresponding semantic interpretations are defined as low-amplitude, medium-amplitude, and high-amplitude, respectively, as shown in Figure 8. The top orange area represents high amplitude, the middle white area represents medium amplitude, the bottom blue area represents low amplitude, and the purple line represents the interval predicted value of the model. The experimental results show that the value corresponding to the semantic “low amplitude” is 0, the value corresponding to “medium amplitude” is 11.4, and the value corresponding to “high amplitude” is 26.3. From Figure 8, we can obtain not only the predicted values of the time series data but also the prediction intervals; in addition, we can obtain the semantic interpretation of the predicted values, which is helpful for people to apply in practice.

## 4. Conclusions

To construct interpretable models and address the issue of previous research focusing only on the time-domain features of time series data and neglecting frequency-domain features, this paper mainly constructs a time series prediction model, WE-HFCM, for non-stationary time series. The WE-HFCM model is designed to offer both interpretability and high prediction accuracy. It stands out for its unique features, such as using a double decomposition strategy to extract time series features. This strategy combines and exploits the advantages of wavelet and EMD decomposition to extract multiple adequate time series features. We then construct high-order fuzzy cognitive maps based on these features and use the ridge regression algorithm to continuously learn and determine the optimal model. Finally, we apply the WE-HFCM model to predict stationary and non-stationary time series, comparing the results with those of the ARIMA and LSTM models. The experimental results, obtained through rigorous validation, demonstrate the superiority of the WE-HFCM model; it has a 45% higher accuracy than the ARIMA model, about a 35% higher accuracy than the SARIMA model, and a 16% higher accuracy than the LSTM model in predicting stationary series. In the prediction of non-stationary series, the WE-HFCM model’s accuracy is 69% higher than that of the ARIMA and SARIMA models, providing a robust solution for time series prediction.

## Figures and Tables

**Table 1:** Data set partitioning.

Dataset	Total Length	Training Set Length	Validation Set Length	Test Set Length
open-price	500	319	79	102
sunspot	289	177	44	67
min-temp	800	448	112	240
windspeed	1000	640	160	200

**Table 2:** Model parameter.

Model	Main Parameters	Explanation
Wave	*N*	Number of WD levels
HFCM	*n*	Number of HFCM nodes
	α	Ridge regression parameters

**Table 3:** Comparison of the experimental results of four models.

Dataset	Model	RMSE	MAE	MAPE
wind-speed	WE-HFCM	*0.509655*	*0.430687*	*12.18910*
	ARIMA	2.887541	2.618241	80.83926
	LSTM	***0.245350***	***0.201483***	***5.843880***
	SARIMA	2.035321	0.463132	16.63178
open-price	WE-HFCM	*1.287810*	*1.056692*	*0.921886*
	ARIMA	128.4047	128.3749	99.96622
	LSTM	***0.614700***	***0.465886***	***0.388289***
	SARIMA	8.183435	7.234018	7.234018
min-temp	WE-HFCM	***3.086318***	***2.414340***	***22.02933***
	ARIMA	8.039095	6.946333	65.90470
	LSTM	*4.204377*	*3.418290*	31.70666
	SARIMA	5.173325	4.050270	*4.050270*
sunspot	WE-HFCM	***16.82442***	***11.45599***	*28.79121*
	ARIMA	55.01083	39.21089	154.4593
	LSTM	62.51576	48.02626	218.0822
	SARIMA	*21.99944*	*16.3178*	***0.463132***

The second best performance result is indicated in italics. The best-performing result is represented in bold and italics.

**Table 4:** Results of RMSE comparison in the ablation experiment.

	Model	WE-HFCM	Wave-HFCM
Dataset			
open-price		***1.287810***	1.984186
sunspot		***16.82442***	20.99618
min-temp		***3.086318***	3.389726
wind-speed		***0.509655***	0.520299

The second best performance result is indicated in italics. The best-performing result is represented in bold and italics.

**Table 5:** RMSE of WE-HFCM on different subsets.

Dataset	Stage	RMSE
wind-speed	all	0.509655
	training	0.509846
	validation	0.473800
	test	0.536034
open-price	all	1.287810
	training	1.238265
	validation	1.391657
	test	1.343341
min-temp	all	3.086318
	training	3.024576
	validation	3.123155
	test	3.181652
sunspot	all	16.82442
	training	15.22556
	validation	13.92508
	test	21.92508

## Data Availability

The raw data provided in the study can be obtained from public databases. We have saved the data used, and they can be accessed at https://gitee.com/gaz123816/second-hand-trading-platform/tree/master/ (23 October 2024).

## Abbreviations

The following abbreviations are used in this manuscript:

WD	Wavelet Decomposition
FCM	Fuzzy Cognitive Maps
HFCM	High-Order Fuzzy Cognitive Maps
LSTM	Long Short-Term Memory
CNN	Convolutional Neural Network
TCN	Temporal Convolutional Network
EMD	Empirical Mode Decomposition
HF	High-Frequency
LF	Low-Frequency
IMF	Intrinsic Mode Functions
RNN	Recurrent Neural Network
ARIMA	Autoregressive Integrated Moving Average
